# Employing Polymer and Gel to Fabricate Scaffold-like Cancellous Orthopedic Screw: Polycaprolactone/Chitosan/Hydroxyapatite

**DOI:** 10.3390/gels11010028

**Published:** 2025-01-02

**Authors:** AmirHossein Badami, Javad Esmaeili, Hasan Mirtalaie

**Affiliations:** 1 Department of Mechanical Engineering, Najafabad Branch, Islamic Azad University, Najafabad 8543131, Iran; amirbadami@smc.iaun.ac.ir; 2 Department of Tissue Engineering, TISSUEHUB Co., Tehran 1956854977, Iran; ja_esmaeili@yahoo.com; 3 Department of Applied Science, UQAC University, Quebec, QC G7H 4V8, Canada

**Keywords:** orthopedic screw, cancellous bone trauma, scaffold, tissue engineering, cell migration, biomaterial

## Abstract

Using metallic/polymeric orthopedic screws causes cavities in bone trauma after the attachment of broken bones, which prolongs the healing. Yet, it remains unknown how to overcome such a challenge. The main aim of this research was to use both polymers and gels to fabricate and study a new PCL/chitosan/hydroxyapatite scaffold-like orthopedic screw for cancellous bone trauma. This screw, because of its low stiffness and its scaffold-based matrix (due to the gel part), can facilitate bone healing. Different concentrations of PCL (60–95% *w/v*) and chitosan (0–5% *w/v*) were blended according to the Response Surface Methodology using the Central Composite Design. The screws were fabricated using the freeze-drying technique. The screws were assessed mechanically, physically, and biologically (cell viability, cell attachment, DAPI, ALP staining, and Alizarin Red staining), and in vivo (a rat subcutaneous implantation model). Based on the results, screws depending on the PCL and gel content depicted different but notable mechanical behavior (10–60 MPa of compressive strength and 100–600 N force). The gel part could affect the physical properties of screws including water uptake (120%), degradation (18% after 21 days), porosities (23%), and mechanical strength (elastic modulus = 59.47 Mpa). The results also demonstrated no cytotoxicity towards MC3T3 cells (>80% cell viability) with good cell attachment, cell concentration, and mineralization (>90%) that was justified by the gel content. The results also showed good in vivo biocompatibility. To sum up, fabricated scaffold-like screws with gel content can be a good candidate for cancellous-bone-based orthopedic purposes. However, more in vitro and in vivo studies are required to optimize the PCL:gel ratio.

## 1. Introduction

Fixation devices are widely employed to repair bone fractures. These implants are made of biocompatible materials such as cobalt, stainless steel, titanium, and alloys like CoCrMo and Ti-6Al-4V [1]. It is important to note that medical implants (interference screws, posts, spiked washers, or staples) are more rigid than bone tissue and contribute to stress shielding phenomena that result in a reduction in bone density, which is named osteopenia [2]. Osteoporosis occurs when an implant removes the normal stress from the bone.

A reduction in bone density makes bones weak and brittle and enhances the likelihood of bone fractures, even with minimal trauma [3]. Orthopedic metallic screws (e.g., stainless steel, magnesium) are known as one of the most employed screws to fix fractured bone species [4]. The biological relationship between the surrounding host tissue and the orthopedic implant may have a significant effect on the clinical outcome [5]. Several efforts have been made to control stress shielding in the case of using orthopedic metallic screws.

For instance, a reduction in the density of the implant has been introduced as an effective method [6]. In this regard, the implants are manufactured with different porosities [7]. However, it has been reported that porous metallic implants still cause mechanical mismatch and stress shielding [8]. Al-Tamimi et al. proposed another strategy to reduce (not remove) stress shielding: changing and optimizing the topology of the implant. [9]. Changing the materials used in some cases showed significant results. For example, cement has been used to study its effect on stress shielding, and, interestingly, cement has a high stress shielding, but it is still lower that of than metal implants [10]. Implanting biodegradable polymers has been another promising approach to decrease stress shielding in comparison with metallic implants [11]. Biodegradable materials demonstrated a lower stiffness and Young modulus than metallic implants [12]. The main benefit of biodegradable-material-based implants is that, as the implant degrades, its mechanical properties decrease, so the level of load on bone is reduced, which means the stress shielding is better controlled [11].

There are several studies that have reported on the fabrication of biodegradable orthopedic screws. For instance, biodegradable-magnesium-based implants showed good results with a Young’s modulus close to that of cortical bone in contrast with the orthopedic metallic screws [13]. Employing polymers expanded the options to fabricate biodegradable orthopedic screws [14]. Weiler and his colleagues compared six types of biodegradable orthopedic screws with titanium screws for Anterior cruciate ligament reconstruction fixation [15]. They reported that biodegradable orthopedic screws, despite creating low stress shielding, showed low torque at failure. Therefore, optimization and long in vitro and in vivo studies were recommended. Moreover, biodegradable orthopedic screws start degradation during bone healing and there is no need for a second injury for screw removal. Furthermore, a reduction in the stiffness of the orthopedic screw will decrease the level of stress shielding.

Tissue engineering brought promising strategies in tissue repair and replacement using biodegradable and biocompatible scaffolds [16]. The scaffolds must be porous to let cells migrate through the whole scaffold along with nutrition diffusion [17]. The mentioned features enhance the rate of healing. Many studies have reported the use of polymeric scaffolds for bone tissue regeneration (reviewed nicely by Shubham et al. [18]). Polycaprolactone (PCL) and chitosan are two well-known biomaterials and their blending in various ratios was employed for bone tissue engineering. PCL is a biocompatible polymer with a slow rate of degradation, while chitosan has a fast biodegradation despite its high biocompatibility. Chitosan has been reported to increase wettability and permeability, accelerate PCL hydrolytic degradation, and improve PCL cell recognition sites [19]. Nanohydroxyapatite (Ca_10_(PO_4_)_6_(OH)_2_) is one of the bioceramics that has properties similar to those of the mineral components of natural bone (calcium). This substance helps in bone induction and cell proliferation. A PCL/chitosan/Nanohydroxyapatite blend has demonstrated a high potential for bone tissue engineering. For instance, Xiao et al. [20] proved that the prepared PCL/chitosan/Nanohydroxyapatite scaffold has good potential in increasing the cell–cell and cell–scaffold interactions. Another study by Rashid Mad Jin et al. reported that the PCL/chitosan/hydroxyapatite scaffold was nontoxic to human skin fibroblast cells (HSF 1184) and had notable cell attachment [21]. Sani and his colleagues fabricated a scaffold made of these biomaterials and, based on their results, the prepared scaffolds increased mineralization and cell proliferation with high viability [22].

To the best of our knowledge, there is no study focusing on using both natural and synthetic biomaterials to develop a scaffold-like cancellous orthopedic screw. The idea behind our study is to fabricate biodegradable orthopedic screws made of polymers and gel (PCL/chitosan/Nanohydroxyapatite) with appropriate porosity, which is hypothesized to act as both screw and scaffold. We believe that the scaffold-like biodegradable orthopedic screws can be a good candidate to cause faster bone repair under lower stress shielding for cancellous bone trauma. In this regard, the main aim was to fabricate a PCL/chitosan/hydroxyapatite scaffold-like screw (PCHSS) and focus on its in vitro evaluation.

## 2. Results and Discussion

A variety of techniques may be used to create scaffolds with the correct features, and similar techniques can also be used to create orthopedic screws with the proper attributes. Free-drying has always been a common cutting-edge method to create scaffolds with the right porosities. For instance, a porous scaffold was created in a study by combining and crosslinking collagen and PCL solution and then freeze-drying it [23]. A common method in scaffold construction is freeze-drying, also known as lyophilization, which is the process of freezing material and then lowering the ambient pressure to enable the frozen water to sublimate straight from the solid phase to the gas phase. This technique is especially useful for maintaining the functional and structural integrity of both synthetic and biological scaffolds [24]. In another study, scientists used the same approach to fabricate a biofunctional magnetic scaffold based on new phosphorylated PCL combined with gelatin [25]. In another study, a chemical grafting technique was used to create PCL-ZA that has been functionalized with zoledronate (ZA). The porous PCL-ZA/gelatin scaffold was created using the freeze-casting technique after adding gelatin to the PCL-ZA polymer solution [26]. It can be concluded that freeze-drying can also be employed to manufacture orthopedic screws [17]. 3D bioprinting is another technique in manufacturing scaffolds and, specifically, it has been used to make an orthopedic screw. The introduction of additive manufacturing to the biomedical industry has facilitated the creation of intricate, adaptable, and repeatable orthopedic implants. The limits of traditional metallic screws are overcome by the layer-by-layer deposition of biodegradable polymer used in the creation of porous orthopedic screws, which promises progressive breakdown and full metabolic resorption. Dhandapanic used 3D printing to fabricate an orthopedic screw [23], while, in our study, a mold was made using the same cancellous screw, which produced threads with the same sharpness; one of the primary issues in 3D printing was the sharpness of threads, which is impacted by the diameter of the nozzle.

### 2.1. RSM Statistical Study

In this research, the CCD model of RSM was employed to study various percentages of PCL and chitosan concentration by Design Expert© 12.0 (Stat-Ease, Minneapolis， MN, USA) software 12.0 to fabricate a scaffold-like orthopedic cancellous screw and optimize the process factors. PCL concentration was considered between 60 and 95% *w/v*, while chitosan concentration ranged between 0 and 5% *w/v*. HA concentration, as the mineral source for bone regeneration, was equal to 2% (based on the PCL content) in all groups. The main aim of the RSM study was to compare the mechanical and physical behavior of all groups. Mechanical behavior, compressive strength (CMS), and Bending Modulus (BM), as responses, are considered as two main factors to compare the fabricated screws mechanically. The responses were examined via analysis of variance (ANOVA). Table 1 summarizes the results.

Table 1 shows the governed equations for CMS and BM. These equations present the relationship between the responses and the concentration of PCL and chitosan. The reliability of a model is usually justified via the *p*-value, which should be lower than 0.05 to conclude that the model fitting the experimental data is valid and significant [27]. As can be seen, the *p*-value for both equations was fewer than 0.0001, which proves the significance of both equations. R^2^ ≥ 0.6 proves the validity of a model [28]. Furthermore, the R^2^ value as the coefficient of determination was 0.94 for both equations. The high value for R^2^ suggests that an acceptable correlation exists between the values predicted by the CCD model and the observed values [29]. The predicted R^2^ (Pre. R^2^) for both models (0.91 and 0.92) was close to the adjusted R^2^ (Adj. R^2^) equal to 0.93 for both models. Adequate precision (AP) compares the range of the predicted values at the design points to the average prediction error, where a ratio higher than 4 is desirable, and, for both models, it is more than 27 [30]. These results confirm that there was a good agreement between the predicted and experimental values including most of the responses.

**Figure 1:**
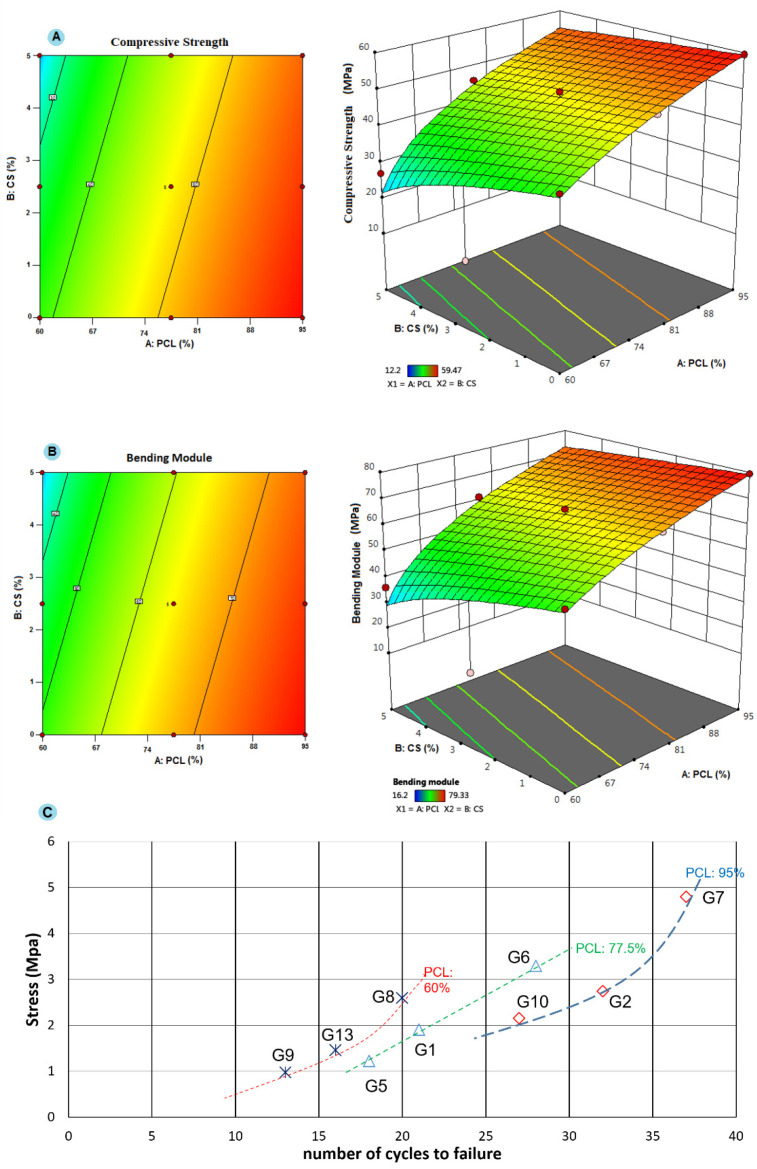
2D and 3D contour plots for the effect of PCL and gel concentration on the mechanical behavior, (**A**) compressive strength, and (**B**) Bending Modulus. Red color indicates a high level of variable and blue color indicates a low level of variable. These diagrams show how changes in the concentration of PCL and gel alter the level of the main parameters. (**C**) S-N diagram obtained from fatigue test which shows how a screw withstands cyclic compressions and strengthens (each line indicates the groups with the same PCL content). (Group [PCL-Chi-HA]: G1 [77.5-2.5-1.55], G2 [95-2.5-1.9], G5 [77.5-5-1.55], G6 [77.5-0-1.55], G7 [95-0-1.9], G8 [60-0-1.2], G9 [60-5-1.2], G10 [95-5-1.9], G13 [60-2.5-1.2]).

Figure 1 depicts how the concentration of PCL and gel (as independent factors) affects ME and BM (as dependent factors). Considering the contours in Figure 1A, increasing the concentration of PCL enhanced the ME, while increasing the gel content affected the ME negatively. Based on Figure 1B, a similar trend was observed for BM. It can be concluded that increasing the gel content reduces the mechanical behavior of the final screws. However, it can be predicted that a gel concentration of less than 2.5% does not affect mechanical behavior notably. Considering PCL, a concentration lower than 90% seemed to result in lower CMS and BM.

Human bone tissues have different mechanical qualities based on things like age, gender, and where the bone is located [31]. Bone tissues’ complicated mechanical behavior makes it intriguing to examine because of the delicate blend of organic and inorganic components that make up these tissues.

It is crucial to consider factors like elasticity, stiffness, strength, and toughness to comprehend the mechanical characteristics of bone tissues. When external pressures are applied and then released, bones with high elastic properties can deform and then reform. On the other hand, stiffness explains how bone resists deformation when a force is applied [32].

Moreover, bone tissues in each part of the body have their specific mechanical properties. More importantly, trabecular bone, also known as cancellous or spongy bone, can be thought of as a highly porous material having anisotropic mechanical characteristics at the visible level (i.e., the scale at which several trabeculae and intervening pores are concurrently observable, generally 5–10 mm). The apparent-level mechanical characteristics of trabecular bone are principally governed by its greater porosity in comparison to cortical bone. The architectural configuration of the trabecular network and the tissue-level characteristics of the individual trabeculae are examples of bone quality features that provide less significant but still significant contributions to the apparent-level behavior [33].

It has been reported that the compressive modulus of cancellus bone ranges between 10 and 157 MPs. This wide range is justified by the location of the bone. Results from our research showed compressive strength in the range of 20–60 MPs depending on the PCL and gel concentrations. The proximal tibia bone showed a compressive strength in the range of 4–430 MPs depending on age, sex, and health condition [34].

The purpose of fatigue testing is to count the number of load cycles necessary to cause a material to fail, as well as to assess the decline in stiffness and strength of the material under repeated loading. Considering the fatigue test (Figure 1C), it can be concluded that the screw with 95% of PCL and 0% gel showed a higher resistance indicated by 37 cycles. The least resistance was attributed to the screw with 60% of PCL and 5% of gel. From the viewpoint of PCL content, increasing the PCL concentration resulted in more cycles, as if, by increasing the PCL content from 60 to 95%, the number of cycles enhanced from 20 to 37. In contrast, gel content showed an inverse effect, as if, by increasing the gel content from 0 to 5%, the number of cycles reduced from 37 to 27 (95%-based screws), from 28 to 18 (77.5%-based screws), and from 20 to 13 (60%-based screws). These results are in line with those obtained from the compression test.

Experimental research was conducted to compare the fatigue characteristics of trabecular bone tissue and similarly sized cortical bone specimens from the human tibia. The results revealed that trabecular specimens had significantly lower moduli and lower fatigue strength than cortical specimens, despite having higher mineral density values [35], and this is while the previous studies indicated a fatigue strength of 7 MPa after 10^7^ cycles [36]. A porous scaffold for the regeneration of articular cartilage was the subject of fatigue research by Vikingsson et al. [37]. According to fatigue experiments, mechanical loading causes the dry scaffold to collapse after fewer deformation cycles than when it is submerged in water. So, it can be inferred that, when the screws are implanted, higher fatigue strength can be obtained due to the penetration of water into the screw, and this provides a true representation of the mechanical performance. In another study, Senatov developed 3D-printed bone replacement PLA and PLA/15% wt. HA porous scaffolds and reported that height loss, pore collapse, delamination, bending and shearing of printed layers, crack growth, and propagation during cyclic loading were observed [38]. Similarly, the screws after the cycles showed an increase in the length and deformation of threads.

### 2.2. Morphology and Surface Analysis

**Figure 2:**
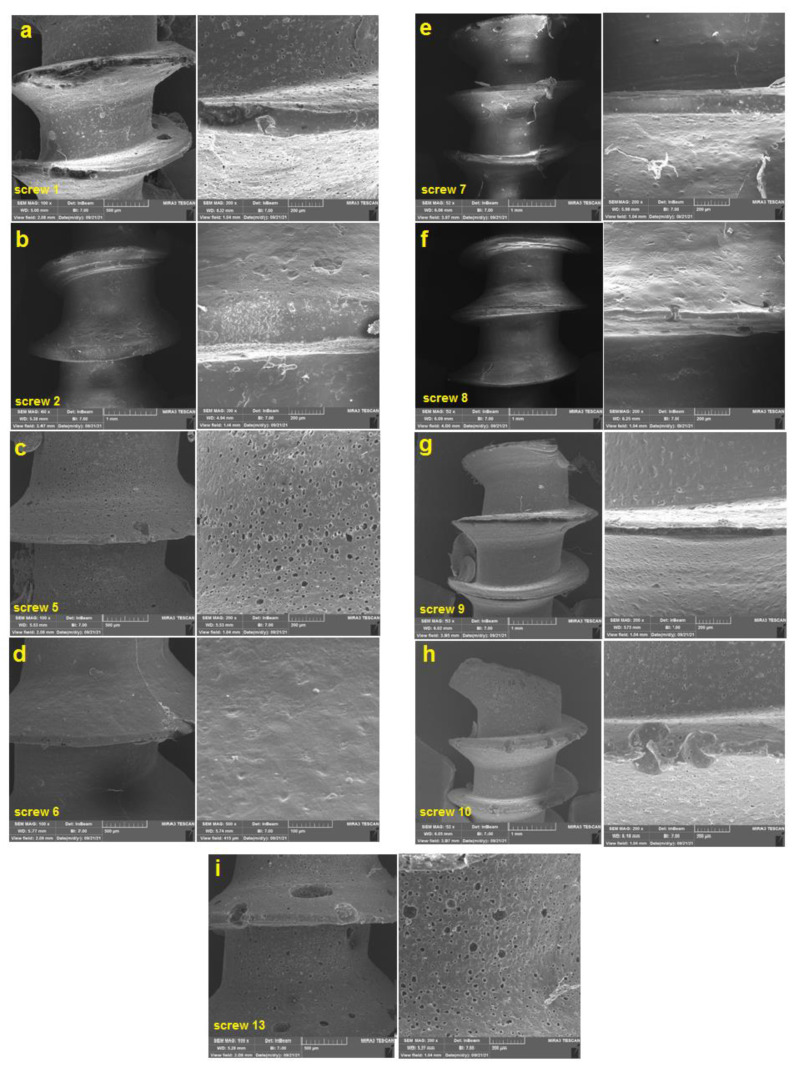
Scanning electron microscopic (SEM) images for PCL: chitosan ratio of (**a**) 77.5:2.5%, (**b**) 95:2.5%, (**c**) 77.5:5%, (**d**) 77.5:0%, (**e**) 95:0%, (**f**) 60:0%, (**g**) 60:0%, (**h**) 95:0%, (**i**) 60:2.5%. Each screw was in a suitable shape similar to the real screw. The threads were formed continuously. The scale bar was 500 μm and 200 μm. (Group [PCL-Chi-HA]: G1 [77.5-2.5-1.55], G2 [95-2.5-1.9], G3 [77.5-2.5-1.55], G4 [77.5-2.5-1.55], G5 [77.5-5-1.55],G6 [77.5-0-1.55], G7 [95-0-1.9], G8 [60-0-1.2], G9 [60-5-1.2], G10 [95-5-1.9], G11 [77.5-2.5-1.55], G12 [77.5-2.5-1.55], G13 [60-2.5-1.2]).

A scanning electron microscopic technique was employed to study the surface and physical structure of the fabricated screws (Figure 2). As Figure 2 depicts, screw numbers 2, 6, 7, and 8 had a similar surface morphology. According to Table 1, screw numbers 6, 7, and 8 were completely made of PCL (77.5, 95, and 60%, respectively), and the gel content was equal to zero in their formulation. Screw number 2 was made of 95% PCL and 2.5% chitosan but had a surface similar to screws 6, 7, and 8 (smooth surface without high porosity). It seems that the presence of gel did not affect the surface morphology of screw 2 notably.

The surface of screw numbers 1, 5, 9, 10, and 13 were similar to each other in the viewpoint of surface morphology and porosity. All screws had a porous surface with a large number of holes in distinct diameters. The porosities were scattered over the surface of the screw shaft and threads. Screw 13 and screw 5 had larger porosity than the others. The smallest porosity belonged to screw 10. Screw 9 depicted a low distribution of porosities. The threads in all screws were formed appropriately. Large cavities were reported in threads of screws containing gel. It can be concluded that the presence of gel increases the porosity of screws.

The presence of these porosities is a critical feature in scaffolds [39]. The main duty of porosities is the transformation of nutrients to all parts of the scaffolds including the inner and central zones [40]. Moreover, they help cells to proliferate, penetrate, and migrate. They are also important for tissue growth and vascularization [41]. Considering bone trauma, pieces of bone are stuck together using orthopedic implants including screws. It would be interesting if a kind of scaffold is used instead of common screws. It is hypothesized that a scaffold-like screw will accelerate bone repair, and bone regeneration happens faster than with current remedies. When an SBOS with suitable porosities and biological characteristics is employed to connect pieces of bone, it can act as a scaffold simultaneously. The porosity of the scaffold-like orthopedic screw helps cell infiltration, nutrient and waste exchange, and mechanical properties. Therefore, understanding the extent to which porosity impacts bone tissue engineering is essential. In this regard, when the scaffold is implanted and cells start to migrate, tissue integration and regeneration within the scaffold start. During this process, the diffusion of oxygen, nutrients, and growth factors starts from the beginning.

Furthermore, porosity impacts the mechanical properties of the screw, as changes in the size and percentage of porosity cause a high or low elastic modulus. Therefore, it is crucial to find an appropriate balance between porosity and mechanical properties [42].

It would speed up the clinical reunion of the fractured bone ends to introduce interconnectivity across the orthopedic screws. It was claimed that interconnected pores (300 m) that were comparable to natural bone tissue prevented the stress-shielding effect without sacrificing mechanical strength [23]. In the case of common orthopedic screws such as orthopedic metallic screws and biodegradable orthopedic screws, due to the lack of porosity, cells have no chance to penetrate the screw.

On the one hand, in the case of orthopedic metallic screws, cells obviously cannot migrate into the screws, and the theory of using a screw as a biological environment for cell growth is not valid. Cells can start proliferation and migration just after screw removal, which, in this regard, takes a long time to fill up the cavities left by the screw [43]. On the other hand, in the case of using biodegradable orthopedic screws, cells can only proliferate and migrate when the screw starts degrading; in this regard, cells have no 3D biological environment to attach to, proliferate, and migrate. However, it has been reported that it takes a long time (e.g., more than 2–3 years) to have the screws degraded [44].

Regarding SBOS, the engineered porosities and using chitosan and PCL with acceptable biological properties raise the chance of faster bone regeneration. Several studies have reported the qualification of chitosan and PCL for bone tissue engineering [45,46]. Their combination also revealed good results for bone regeneration [47]. Rashid Mad Jin and his colleagues fabricated a scaffold made of PCL (5% *w/v*) and chitosan (3% *w/v*) for tissue engineering purposes [21]. They reported a high potential for this scaffold with acceptable porosities. Changes in the concentration of PCL and gel influence the level of porosity. Chandramohan et al. [48] used PCL (5% *w/v*)/chitosan (1.5% *w/v*) loaded with zinc nanoparticles for bone tissue engineering.

It was reported that the created porosities within the scaffold matrix and its good biological features promoted osteoblast differentiation. In another study, Sharifi and her colleagues [47] reported the usage of PCL (12.5%)/chitosan (5–15%) to create a nanofiber-based scaffold for bone tissue engineering. Similarly, they reported that the cells showed good cell attachment and proliferation through the scaffolds.

### 2.3. Mechanical Behavior

As previously mentioned, the PCL/chitosan-based scaffold proved its potential for tissue regeneration. An interesting point was that the ratio plays a vital role in making them suitable for soft or hard tissues. Regarding bone tissue engineering, it is mandatory to create a scaffold with high mechanical properties while having good biological characteristics [49]. PCL, as a synthetic polymer, has always shown good mechanical properties in tissue engineering, but it suffers from a lack of biological features and cell attachment due to low hydrophilicity and cell signal recognition [50]. However, surface modification and combination with natural polymers like chitosan, gelatin, and sodium alginate using nanoparticles are approaches to create PCL-based scaffolds for tissue engineering purposes. It must be mentioned that PCL (in low concentration) combined with natural biomaterials can also be employed for soft tissue engineering such as lung [51].

**Figure 3:**
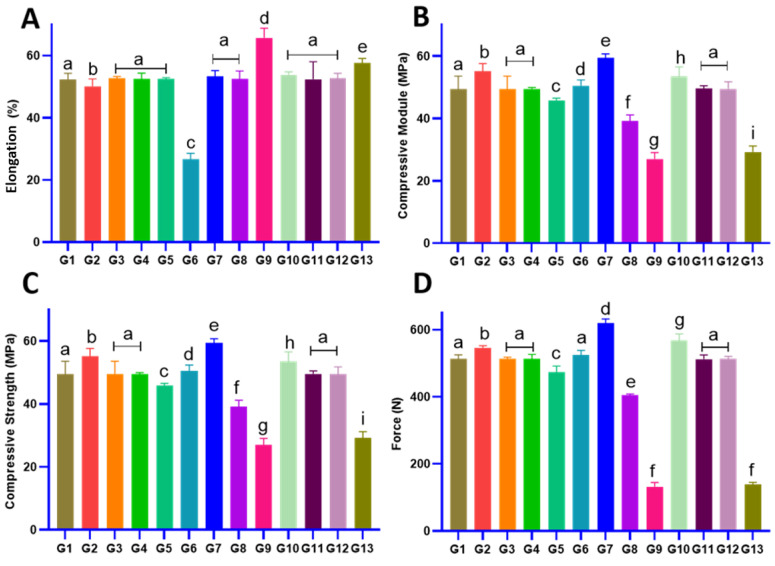
Mechanical behavior for all screws: (**A**) elongation, (**B**) compressive strength, (**C**) Bending Modulus, (**D**) maximum force at the break (same letters mean no significant difference, distinct letters mean significant difference). In general, increasing the PCL content showed an increase in mechanical strength, while increasing the gel content showed an inverse effect. (Group [PCL-Chi-HA]: G1 [77.5-2.5-1.55], G2 [95-2.5-1.9], G3 [77.5-2.5-1.55], G4 [77.5-2.5-1.55], G5 [77.5-5-1.55],G6 [77.5-0-1.55], G7 [95-0-1.9], G8 [60-0-1.2], G9 [60-5-1.2], G10 [95-5-1.9], G11 [77.5-2.5-1.55], G12 [77.5-2.5-1.55], G13 [60-2.5-1.2]).

Increasing the PCL content is assumed to enhance mechanical strength. Figure 3 depicts the mechanical behavior of all fabricated screws focusing on elongation, CMS, BM, and the maximum force at break (MFB) (Gi: screw i, i = 1, 2, …, 13). Considering elongation (Figure 3A), screws 1, 3, 4, 5, 11, and 12 comprised 77–95% PCL, and 2.5% chitosan showed similar elongation (52%) (*p* > 0.05), with no significant difference with screw 7 (52%) and screw 8 (53%), which are made of 95 and 60% pure PCL, respectively. The higher and lower elongation belonged to screw 9 (64%) and screw 6 (26%), respectively. In general, the addition of gel enhanced the elongation of all screws.

Considering the CMS (Figure 3B), increasing the PCL content (gel equaling zero) caused a significant enhancement in CMS (*p* < 0.05). Increasing gel content in screws with 60, 77.5, and 95% PCL content caused a significant reduction in CMS from (*p* < 0.05). For instance, CMS reduced from 39 MPa to 27 MPa for 60%-PCL-based screws. The most (59 MPa) and least (12 MPa) CMS were attributed to screw 7 (95% PCL: 0% chitosan) and screw 9 (60% PCL: 2.5% chitosan). A similar trend was observed for Bending Modulus as the high and low BM belonged to screw 7 (79 MPa) and screw 13 (16.2 MPa), respectively. In the case of the MFB, no significant difference was observed between screws 1, 3, 4, 6, 11, and 12 (500 N). No significant difference was observed between screw 2 (546 N) and screw 6 (524 N). The highest MFB belonged to screw 7 (619 N) and the least MFB was recorded for screws 9 and 13 (130 and 138 N, respectively), which contained 60% PCL and 2.5–5% chitosan.

Considering Figure 1 and Figure 3, it can be concluded that PCL plays a vital role in boosting the mechanical behavior of the screw. Reducing the PCL content from 95% to 60% showed a significant reduction in the mechanical properties (EM, BM, and MFB). The justification for this reduction turns back to the low density of the primary polymer solution [52]. In the case of screws made of PCL and gel, increasing the gel content caused lower mechanical behavior. Comparing the pure PCL scaffolds to the PCL/gel scaffold, the presence of gel results in low mechanical behavior. The rationale behind this is attributed to the low mechanical behavior of the gel [53]. Previous studies reported the same approach in the case of gel/PCL-based scaffolds. Considering lung tissue engineering, the gel content increased (PCL:chitosan; 1:3) to reach a high elongation and low stiffness [51]. However, chitosan, owing to its good biological properties and its low mechanical behavior, has always attracted the attention of scientists in bone tissue engineering [54]. PCL, due to its high mechanical and biocompatibility properties, is one of the abundant biomaterials and its main function is supporting the matrix mechanically [55].

Regarding the cancellous bone, CMS was reported for cortical bone (100–230 MPa) and cancellous bone (2–12 MPA) [56]. Considering the fabricated scaffolds for bone tissue engineering, the final scaffold depicted distinct mechanical behaviors. For instance, Yedekçi and his colleagues reported CMS equal to 9–24 MPa for PCL-PEG-PCL/strontium scaffold containing less than 18% PCL [57]. In another research paper, a 3D-printed PCL-based scaffold was fabricated with 18 MPa mechanical strength that was comparable to trabecular bone (12–120 MPa) [58]. Regarding PCHSS, a CMS of 10–60 MPa was recorded and the high content of PCL was considered as the main rationale for higher mechanical behavior. They reported that the lower the biomaterial content, the higher the porosity in the scaffold structure, which probably affects the mechanical properties negatively [59]. Focusing on the main function of PCHSS, it is mandatory to fabricate PCHSS with appropriate CMS, BM, and MFB related to the defective bone. Therefore, it is possible to fabricate more PCHSS with various PCL concentrations. Dhandapani and his colleagues fabricated a Polylactic acid-based orthopedic screw using 3D bioprinting. They reported that the screw with 45% Polylactic acid had CMS and MFB equal to 24MPa and 482 N, respectively [23]. This is while the PCHSS screw with 60% PCL content (0% gel) had CMS and MFB equal to 39 MPa and 405 N, respectively. Moreover, other PCHSS had higher and fewer CMS and MFB in comparison to the Polylactic acid-based orthopedic screw. In another study, Sadeghi et al. fabricated a poly (L-lactic acid)-based screw using a molding approach [60]. Based on the results, the MFB ranged between 208 and 263 N. The justification behind these differences turns back to the employed biomaterial, geometry of the screw, and additives such as hydroxyapatite. Moreover, fabrication techniques and thermal and mechanical processing conditions can change the mechanical properties [56]. In this regard, it has to be pointed out that PCHSS are supposed to act as an orthopedic screw and scaffold to accelerate bone regeneration.

### 2.4. Water Uptake, Degradation, and Porosity

One of the main properties of scaffolds is water uptake. During cell culture, water uptake accelerates the attachment, infiltration, and migration of cells into the scaffolds. Water uptake also expands the total porosity and pore size and, finally, causes an enhancement in the internal surface area of the scaffolds [61]. Since PCHSS are supposed to act as scaffolds, they were fabricated in the shape of cancellous screws for orthopedic purposes; having good water uptake is mandatory for having good biological activity.

**Figure 4:**
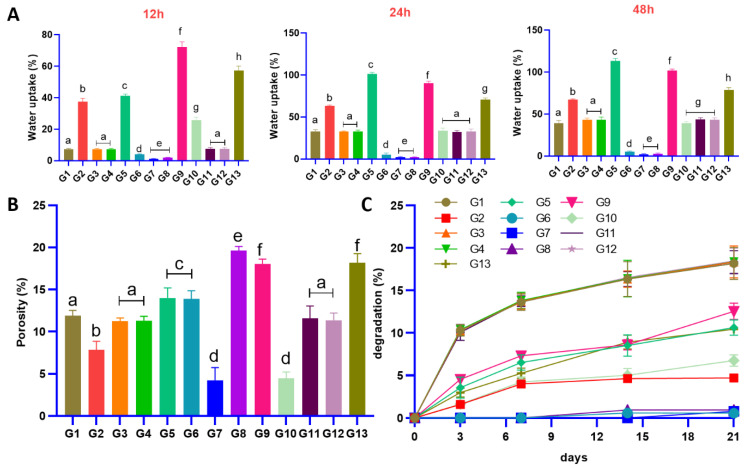
(**A**) Water uptake rate for all screws after 12, 24, and 48 h. Increasing the gel content enhanced the level of water uptake. (**B**) The percentage of porosity for all scaffolds, increasing the gel content, affects the porosity (**C**) and the rate of degradation of screws at 37 °C after 3, 7, 14, and 21 days. The presence of gel directly affects the rate of degradation (same letters mean no significant difference, distinct letters mean significant difference). (Group [PCL-Chi-HA]: G1 [77.5-2.5-1.55], G2 [95-2.5-1.9], G3 [77.5-2.5-1.55], G4 [77.5-2.5-1.55], G5 [77.5-5-1.55], G6 [77.5-0-1.55], G7 [95-0-1.9], G8 [60-0-1.2], G9 [60-5-1.2], G10 [95-5-1.9], G11 [77.5-2.5-1.55], G12 [77.5-2.5-1.55], G13 [60-2.5-1.2]).

As can be seen in Figure 4A, after 12 h, all scaffolds with various PCL and gel showed water uptake. Screws 2, 5, 9, and 13 showed a fast water uptake (*p* < 0.05). Screws 1, 3, 4, 11, and 12, which had the same composition, showed no difference (*p* > 0.05). The highest and least water uptake belonged the screws 9 and 7 (*p* < 0.01). However, no significant difference was observed between screws 7 and 8 (*p* > 0.05). After 24 h, all screws showed a significant increase in water uptake (*p* < 0.05), except screws 6, 7, and 8 (*p* > 0.05).

Screws 1, 3, 4, 10, 11, and 12 depicted the same trend in water uptake during 48 h. In general, the most water uptake was attributed to screw 9 made of 60% PCL and 5% chitosan. It was also concluded that enhancement in gel content increased the level of water uptake. For instance, in the case of scaffolds made of 60% PCL, by increasing the gel content from zero to 5%, the water uptake increased from 2 to 101%. The main reason can be attributed to the high hydrophilicity of chitosan [62]. Furthermore, it can be predicted that increasing the PCL content results in a more dense matrix (low porosity); therefore, the least water uptake ratio belonged to the screws with pure PCL content. Moreover, PCL is known as a hydrophobic biomaterial that depicts low water adsorption [63]. However, a number of methods, such as surface modification and the combination with natural biomaterials, can enhance PCL’s hydrophilicity and water absorption [64]. So, the addition of gel showed appropriate improvement in the water uptake of screws. Last, but not least, water uptake can fill up the gap between bone and screw, which facilitates cell attachment, migration, and proliferation [65].

As can be seen in Figure 4B, all screws depicted various percentages of porosities that prove the porous structure of screws. Notwithstanding these porosities, it is vital to achieve the porosity appropriate to the defective bone matrix. Screw 8 and screw 7 showed high and low percentages of porosity equal to 19.61% and 4.23%, respectively, although no significant difference was observed between screws 7 and 10 (*p* > 0.05). Screws 1, 3, 4, 11, and 12 resulted in nearly the same percentage of porosity (10–12%) (*p* > 0.05). Screws 5 and 6 showed a porosity level of 10% without any significant differences (*p* > 0.05). Screws 9 and 13, with porosity levels of 18% (*p* > 0.05), showed the most porosity after screw 8 (*p* < 0.05). Based on the results, it can be concluded that increasing the gel content affects the porosity. Moreover, increasing the PCL content reduces the porosity.

Porosity is known as another critical property of scaffolds. It helps nutrition to be delivered to the central zones of scaffolds. It also helps cells to migrate and spread over the scaffold, resulting in a uniform cell culture and tissue regeneration [66]. Regarding bone tissue engineering, the porosity and pore size of biomaterial scaffolds play a critical role in bone formation in vitro and in vivo. It has been reported that the level of porosity not only influences the mechanical properties of the scaffolds, but also affects osteogenesis in vivo and in vitro [67]. In the case of PCHSS, this porosity is critical to help cells attach and migrate into the screw and start proliferation while the screw keeps the bone pieces stuck together. The presence of porosity in orthopedic screws can influence the mechanical behavior of the screws too [68]. However, from the viewpoint of biological activities, the created porosities let cells attach to the screw’s wall in the first moments of screw implantation, which is considered as a specific potential for PCHSS screws. It is necessary to distinguish that the pore size and geometry are critical parameters that must be considered in this regard [69]. Lower porosity stimulates osteogenesis by suppressing cell proliferation and forcing cell aggregation. In contrast, higher porosity and pore size results in greater bone ingrowth [67]. To sum up, PCHSS with distinct porosity, according to the desired bone tissue, can be fabricated with appropriate porosity and it is possible to manipulate their size and geometry, which requires further study.

Cells require additional room to fill the entire screw and bone cavity after attaching and occupying the space inside the screw. In this sense, another parameter that is essential to bone regeneration is degradation. Figure 4C reports the in vitro degradation rate (DR) of screws at biological pH = 7 for 21 days. Screws 1, 3, 4, 11, and 12 showed fast weight loss after 3 days and the most DR after 21 days. Screws 6 and 8 did not show degradation until day 14, and screw 7 showed no degradation after 21 days, which is the main justification for their low DR turning back to their pure PCL composition that has an inherently low DR [70]. However, they showed the least degradation. Screw 5 and screw 13 showed similar trends in degradation during 21 days. Interestingly, screw 2 showed no specific degradation after day 14. In general, it can be assumed that screws containing gel depicted high DR because chitosan is highly biodegradable [71]. Increasing the gel content depicted an enhancement in DR.

The rate of bone tissue regeneration must match the rate of scaffold degradation and early mechanical support be provided [72]. It has been reported that the biological degradation of scaffolds in vivo is faster than that in vitro. The reason behind this may be attributed to bone mineralization and biological fluid flow around the scaffold [72]. However, more studies must be carried out to determine the mechanical behavior of scaffolds during in vivo studies. Considering PCHSS, it seems that the modification of the PCL and gel content can lead to a screw with controlled DR. Despite the optimization of the composition, other parameters including crosslinking, fabrication technique, size and geometry, size of the defect, location in the human body, and presence of nanoparticles are other factors affecting DR [73,74].

### 2.5. Hydrophilicity

A hydrophilic scaffold indicates a strong affinity for water and dissolves in water or biological media very easily [75]. Hydrophilicity plays a vital role in altering the rate of degradation and water uptake [76]. Considering PCHSS screws, high hydrophilicity is desired to increase its potential as a scaffold. However, it was reported that hydrophilicity could affect the mechanical behavior of scaffolds in vivo and in vitro [77]. Hence, it is mandatory to consider the level of hydrophilicity for PCHSS fabrication.

Table 2 illustrates the contact angle of all PCHSS screws as the main index to compare their hydrophilicity. Screws 6, 7, and 8 owned the largest contact angle equal to 81.87°, 85.86°, and 83.94°, respectively. The reason can be attributed to their composition, as they are made of pure PCL. Based on previous studies, it was reported that a pure PCL scaffold showed a contact angle equal to 96° [63]. The contact angle for screws 1, 2, and 13 was around 79°, and screws 5, 9, and 10 had contact angles equal to 60.57°, 45.24°, and 69.02°, respectively. The smallest and largest contact angles belonged to screw 9 and screw 7.

Comparing the screws, it was observed that the lack of gel increases the contact angle, which indicates low hydrophilicity. In contrast, increasing the gel content reduced the contact angle, indicating high hydrophilicity. Similar results were reported by Sadeghi et al., focusing on the chitosan impact on the hydrophilicity of a PCL-based electrospun scaffold for neural tissue engineering [62]. In this study, the presence of chitosan modified the surface of PCL and its potential in water uptake, which is significant for cell adhesion, proliferation, and migration [64]. Considering PCHSS, after the implantation of the screw, the screw is assumed to uptake a high amount of water to be prepared as a 3D environment for cells. In this regard, the more hydrophilicity, the faster the cell attachment and migration [78]. So, it is mandatory to consider optimized hydrophilicity for an orthopedic screw such as PCHSS. However, other main factors including the mechanical and physical properties must be under control.

### 2.6. Biological Behavior

Apart from mechanical and physicochemical properties, a scaffold must have good biological properties too [79]. This criterion is mandatory for PCHSS screws owing to their dual role as orthopedic screws. The cell viability of PCHSS screws was assessed using MTT assay. Generally, the screws were divided into three types: pure PCL, PCL + 2.5% chitosan, and PCL + 5% chitosan. It is necessary to point out that all screws contain 2% hydroxyapatite (based on PCL weight) as the mineral source for facilitating bone tissue regeneration. The screws containing 95% PCL according to the mentioned classification were nominated for MTT assay as follows: screw 7 (0% gel), screw 2 (2.5% gel), and screw 10 (5% gel).

**Figure 5:**
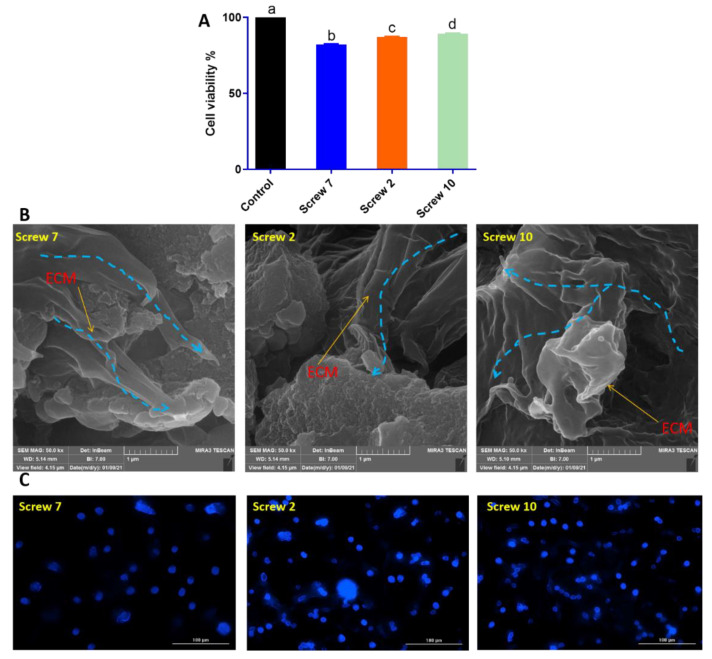
Results for (**A**) cell viability obtained from MTT assay against MC3T3 cell line as an osteoblast precursor cell line for 48 h, (**B**) SEM for cell attachment evaluation, in which the distribution of ECM can be observed, and (**C**) DAPI staining with the scale of 100 μm for three nominated screws containing 95% PCL (screw 7), 95%:2.5% (screw 2), and 95%:5% (screw 10) (same letters mean no significant difference, distinct letters mean significant difference) (blue dashed lines show the direction of ECM movement and spreading).

Figure 5A demonstrates the cell viability of screws 7, 2, and 10 against the MC3T3 cell line as an osteoblast precursor cell line for 48 h. As can be seen, all screws depicted good viability toward the MC3T3 osteoblast cell line. Increasing the gel content enhanced the cell viability from 80 to 90%. This trend can also be predicted for other screws with 60% and 77.5% PCL combined with different gel concentrations. These results prove that the PCHSS screws are biocompatible and show no significant cytotoxicity. Results from SEM analysis (Figure 5B) proved that MC3T3 cells could successfully attach to the screw’s structure. The extracellular matrix (ECM) was formed (red arrows) and spread over the internal structure of the screws (blue arrows). By evaluating the ECM, it can be seen that migration can be confirmed (blue arrows).

It can also be claimed that, by increasing the gel content, more proliferation and migration can be seen. This claim can also be confirmed by results from DAPI staining (Figure 5C). Figure 5C demonstrates the healthy cells with an increase in their population. DAPI estimates were inferred from the results of cell viability and SEM. The biological results proved that the MC3T3 osteoblast cells viability, growth, and proliferation on PCHSS screws can be improved and facilitated by adjusting the PCL/gel ratio. However, it must be mentioned that, by adjusting the porosity, rate of degradation, and addition of mineral sources, these features can be improved too, which needs further research and experiments.

According to previous studies, no cytotoxic effect has been reported for PCL/chitosan scaffolds in bone tissue engineering [80]. For instance, Mad Jin et al. evaluated PCL/chitosan for tissue engineering purposes and reported a high level of biocompatibility for all types of scaffolds, regardless of the ratio of PCL/chitosan [21]. In another study, Semnani et al. studied PCL/chitosan scaffolds for liver tissue engineering [81]. They reported a high potential of scaffolds for biomedical applications. PCL/chitosan scaffold also showed their potential in the tissue engineering of different organs such as lungs [51], bones, nerves, cartilage, eyes, heart, liver, kidney, and skin [19]. To sum up, the fabricated PCHSS are predicted to show good in vitro and in vivo biological behavior. However, more studies are necessary to optimize the best PCL:chitosan ratio to reach a suitable scaffold-like orthopedic screw and also make it a good candidate for clinical trials.

**Figure 6:**
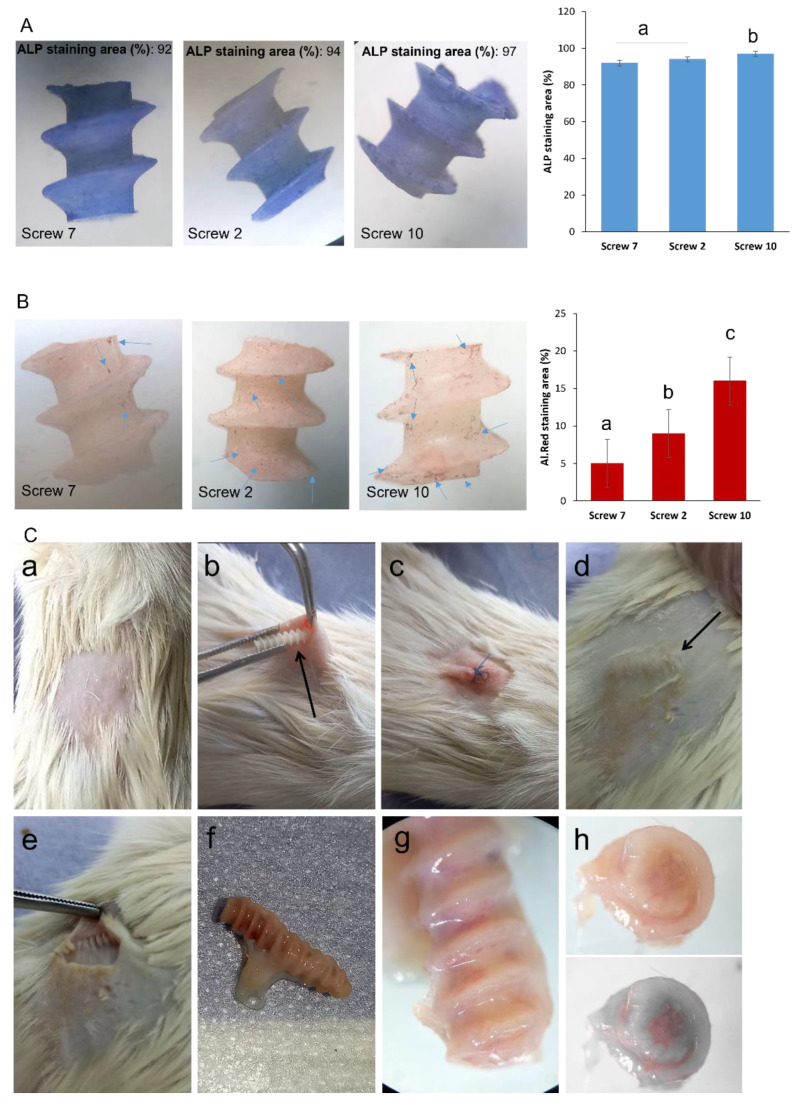
Results of (**A**) ALP staining using MC3T3 cell line as an osteoblast precursor cell line for 14 days, and it can be seen that all groups showed notable (**B**) Alizarin red staining of MC3T3 cells that show more mineralization by increasing the gel content for three nominated screws containing 95% PCL (screw 7), 95%:2.5% (screw 2), and 95%:5% (screw 10). (Same letters mean no significant difference, distinct letters mean significant difference). (**C**) In vivo biocompatibility of the screw 10; (**C**(**a**–**c**)) the screw was implanted on the back of a rat (male, 250 g) for 27 days; (**C**(**d**–**h**)) after 30 days the screw was removed.

The osteogenic potential of the nanocomposite scaffolds was studied using ALP activity as an indicator of early-stage osteocyte differentiation (Figure 6A). According to the data, a high level of ALP content (>90%) was observed for the nominated screws. Another interesting finding was that, by increasing the chitosan content, more ALP deposition was observed, which can be justified by providing a more ECM-like environment with better cell attachment properties. In addition, the presence of HA can be considered a critical factor for higher ALP content. It has been suggested that the presence of HA advances the adhesion, proliferation, and differentiation of cells engaged with bone reconstruction, finally prompting an expanded ALP content in the scaffold. More interestingly, ALP promotes bone formation by degrading inorganic pyrophosphate (PPi), an inhibitor of hydroxyapatite formation, and generating phosphate ions that can combine with calcium ions to form hydroxyapatite [82]. Typically, the matrix mineralization was studied by Alizarin red staining to monitor the calcium deposition in vitro, as indicated by dark red spots after seeding BMSCs (Figure 6B). The results showed calcium mineralization for all screws, and, interestingly, mineralization was higher in screws with gel content.

These results indicated that the fabricated screws in each formulation (pure PCL or PCL/gel) have the potential to cause bone regeneration. However, previous studies confirmed the potential of PCL/gel-based scaffolds in bone tissue reconstruction [83]. These scaffolds are very biocompatible, meaning that the body tolerates them well and can support the growth of new bone tissue. PCL is a biodegradable polymer that provides mechanical strength to the scaffold, while chitosan, derived from crustacean shells, promotes cell adhesion and proliferation [84]. These approaches in the screws help bone regeneration frequently after implantation.

Last, but not least, in vivo biocompatibility was studied via the subcutaneous implantation of screws on the back of a male rat (Figure 6C). As can be seen, the scaffold showed tissue reconstruction after 30 days. First, this finding indicates that the scaffold can be a good candidate for tissue reconstruction and confirms its good ECM-like behavior.

In addition, there was no edema or local infection around the regenerated tissue and surrounding tissues (Figure 6(Ce)). During the study, the rats were monitored and there was no scratching. There was a tight bonding between the regenerated tissue and native tissue, which confirms a kind of integration between the native and regenerated tissues (Figure 6(Cf)). Based on Figure 6(Cg,Ch), it can be concluded that cells could migrate into the scaffold. These results showed that the screws not only have the potential for cell attachment, but they are also degradable and can provide a biological environment for cell migration, cell growth, and cell proliferation.

## 3. Conclusions and Future Perspectives

In this research, an in vitro study was carried out to develop a new scaffold-like orthopedic cancellous screw for cancellous bone trauma injuries. To achieve this, PCL and chitosan were nominated and blended according to the RSM study using the central composite method. Hydroxyapatite was considered equal to 2% (based on the polymer weight) for all screws as the mineral source. PCL and chitosan were employed as the main biomaterials in different concentrations [PCL: 60–95%, chitosan: 0–5%]. The screws were fabricated using the freeze-drying technique. Based on the results, the blending of both PCL and chitosan exhibited good potential in providing good mechanical, physical, and biological properties including biocompatibility and mineralization. In brief, PCL played a vital role in adjusting the mechanical behavior of screws, while chitosan, due to its good biocompatibility features, affected the physical and biological properties. Depending on the injured cancellous tissue in the human/animal body, this formulation can be a candidate for further studies. Screws demonstrated good viability towards MC3T3 osteoblast cells. They also showed that they have the potential for calcium deposition and mineralization. Considering the in vivo results, the nominated screw (screw 10) showed good potential in tissue reconstruction without edema and inflammation. It can be concluded that the fabricated screws could be a good nominee as a biodegradable orthopedic screw.

Based on our hypothesis, this kind of screw will facilitate bone regeneration in cancellous bone trauma where orthopedic screws are employed. Orthopedic screws (e.g., metallic screws) create a cavity after removal, which not only weakens the bone, but also causes it to take a long time to repair. PCHSS screws are a good candidate for orthopedic purposes as their potential can be enhanced under more in vitro and in vivo studies. In this regard, the mechanical behavior, porosity, water uptake, rate of degradation, and biological properties can be optimized. PCHSS screws are predicted to be a good candidate for several options: (i) PCHSS screws can be implanted instead of the metallic screw from the beginning for cancellous bone injuries, and (ii) PCHSS screws can be replaced with a metallic screw after bone healing to facilitate bone regeneration (both cancellous and cortical bones). As an important challenge in the fabrication of scaffold-like screws, making screws with high porosity while having good mechanical behavior can be studied further. Employing a variety of biomaterials and their modifications can create notable distinguished differences in screws. PCHSS are recommended to be studied for their impact on controlling stress shielding during bone healing. However, it can be a good recommendation to find out if no distinguished stress shielding is predicted for PCHSS in comparison with the metallic orthopedic screws.

## 4. Materials and Methods

### 4.1. Materials

Polycaprolactone (PCL, Mw 80,000 Da, Sigma-Aldrich, Darmstadt, Germany), chitosan (CS, Mw of 50,000–190,000 Da, Sigma-Aldrich), hydroxyapatite (Sigma-Aldrich), chloroform (Merck, Burlington, MA, USA), and acetic acid (AA, 100%, Merck) were purchased from the local supplier (TemadKala Co., Tehran, Iran). All the reagents and the materials were of analytical grade.

### 4.2. Design Expert

In this study, Response Surface Methodology (RSM) using Central Composite Design (CCD) was employed to find the optimum formulation to fabricate PCHSS with proper mechanical behavior, scaffold behavior, and high biocompatibility. The main parameters including PCL concentration *“B*” and chitosan concentration “*A*” were evaluated upon the optimized formulation. Three levels, including high (+1), medium (0), and low (−1), were considered for A and B, separately. Based on our literature study focusing on scaffolds for bone tissue engineering, the low and high levels for B were 60% and 90% *w/v*, respectively, and for A they were 0% and 5%. The concentration of hydroxyapatite (HA), as a calcium source, was considered equal to 2% of the PCL content. Table 3 presents 13 runs reported from RSM. Porosity and mechanical behavior were considered as the responses. The measured responses were recorded in the software to provide the governed equations between material composition and the considered response and also to illustrate the relevant graphs.

### 4.3. Preparation of Polymeric Solution

The polymeric solutions were made according to Table 3. The final polymer solution was equal to 5 mL.

**Figure 7:**
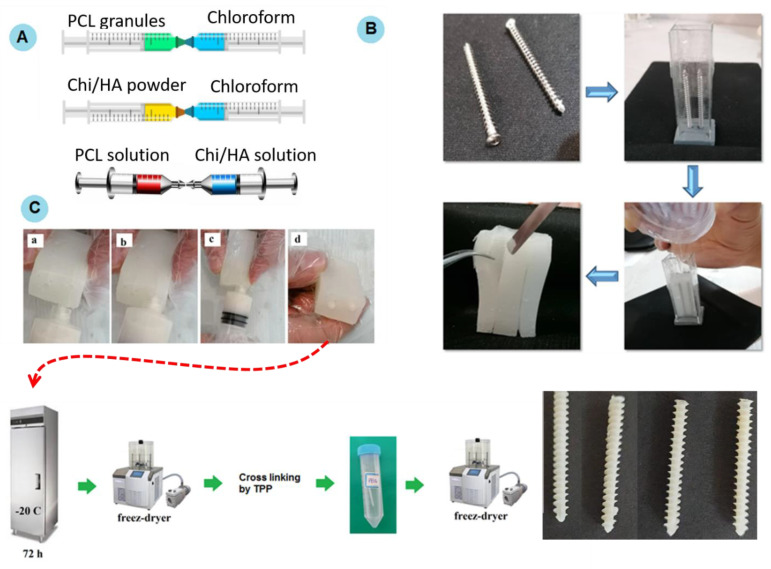
(**A**) Preparing PCl/chitosan/HA blend in three steps including preparation of the PCL solution, Chi/HA (gel) solution, and final polymeric solution. (**B**) Making silicon mold for the cancellous screw using screws with a diameter of 5 mm and length of 6 cm. (**C**) Illustration of PCL/chitosan/HA screw fabrication by injecting the final polymer solution into the fabricated mold.

***PCL solution:*** First, in each run, the PCL granules (according to Table 3) were dissolved in 4 mL of chloroform using two connected syringes (Figure 7A). For example, for run 1, the required PCL for a 5 mL polymer solution was equal to 3.87 g [(5 × 77.5)/100].

***Gel solution:*** Then, chitosan powder (according to Table 3) and hydroxyapatite (2% of PCL content) were mixed and transferred to a syringe containing 1 mL chloroform. To dissolve chitosan powder, 100 μL of 95% *v/v* acetic acid was added and mixed using two connected syringes to reach a uniform solution (Figure 7A).

***Final polymer solution:*** The prepared PCL solution was blended with a gel/HA mixture using two connected syringes. In this step, PCL and gel were viscose liquids, and, by mixing them, the gel was dispersed through the PCL, finally obtaining a uniform gel-like liquid. The volume of the final solution was stored at 4 °C.

### 4.4. Preparing the Screw Mold

To make the screw mold, an orthopedic cancellous screw (length: 6 cm, diameter: 5 mm) was used as the model. Cancellous screws are made of stainless steel and are designed to fix bone segments. In this way, according to Figure 7B, first, the screw was fixed on a piece of dough by pushing 1.5–2 mm of the screw into the dough. Then, using glass slides, the body of the mold was created in a rectangular shape around the screw. Then, 20 g of silicon was mixed with 0.3 g of silicon hardener. Next, the silicone solution was poured into the mold and left at room temperature for 48 h to allow the silicone to harden. Finally, the silicone mold was removed from the glass mold and the embedded screw was detached from the silicone mold.

### 4.5. Screw Fabrication

The obtained polymer/gel solution from Section 2.3 was injected into the screw mold (obtained from Section 2.4) using a syringe at low speed, as shown in Figure 7C. Then, the molds were transferred to a −20 °C freezer for 72 h and then were kept at −80 °C for 48 h. Then, the samples were detached and freeze-dried using a freeze dryer (Arian vacuum, Tehran, Iran) to remove the residual solvent. Sodium tripolyphosphate solution (TPP) was used to crosslink the chitosan content of the screws. To achieve this, the freeze-dried samples were immersed in 1% TPP solution for 2 h. Next, the samples were washed several times with PBS. Finally, the samples were frozen at −80 degrees for 48 h and freeze-dried again. Samples were stored in the refrigerator (4 °C) for the rest of the study. The final dimension of the fabricated screws was 5 mm in diameter and 4 cm in length [17].

### 4.6. SEM

The morphology and surface structure of the PCHSS were studied by scanning electron microscopy (SEM) (Philips XL30; Philips, Eindhoven, The Netherlands) under a 25 kV accelerating voltage after sputtering a 5 nm diameter gold layer on samples.

### 4.7. Mechanical Analysis

A compression test was used to investigate the mechanical properties of PCHSS using a SANTAM mechanical tester (Iran). In this way, each sample was pressed by applying an initial force of 200 N at a speed of 5 mm per minute. The samples (length: 2 cm) were fixed in two fixtures as if the gap between the fixtures was 10 mm and then placed between the load cells. The specimen size was considered according to the ASTM-F2150-19 Standard as if the ratio of high/diameter of the specimen was 10/5 [85]. An average of three measurements was taken for each sample.

Cyclic fatigue testing was performed on screws (3 cm high, 5 mm diameter), to find the effect of formulation on fatigue properties [86]. Cyclic loading was applied on screws using the SFT-600 Santam rotating bending fatigue device. Since there is a rotation for the screw, the maximum bending stress on the specimen surface will be fully reversed. The investigation was conducted in air at a frequency of 0.2 Hz and a strain ratio, R, of 0.5. With specimens examined at strain levels ranging from 0.7% to 3.0%, the a-Nf curves were produced.

### 4.8. Water Uptake

The water uptake study was performed according to the previous studies [87]. The primary weight of the PCHSS was measured (*W*_0_). The samples were then incubated in 10 mM PBS solution in pH 7.4 at 37 °C for 24 h. Then, a Kimwipe was employed to remove the remained liquid from the samples before weighing each sample (*W*_1_). The water uptake of the samples was calculated using Equation (1).

(1) $$\%\;\mathrm{Water}\;\mathrm{uptake}=\frac{W_{t}-W_{0}}{W_{0}}\times 100$$

### 4.9. Contact Angle

The hydrophilicity of PCHSS (the fabricated screws) was assessed by water contact angle measurements using JC2000D3, SZDTA, Shanghai Zhongshan Digital Technology Apparatus Co., Shanghai, China). Thin films (2 × 2 cm^2^) were prepared for each group. In total, 1 microliter of water was dropped on the surface of the film and the contact angle of water was measured after 10 s.

### 4.10. Porosity

The porosity of the screws was analyzed using the liquid displacement technique with pure ethanol [88]. Briefly, a screw (length = 2 cm) was immersed in a graduated test tube containing *V*_1_ mL of ethanol for 10 min. Ethanol is diffused into the pores of the screws. The final volume of the graduated test tube (total volume of the ethanol and screw) was then recorded as *V*_2_. The ethanol-loaded screw was then removed carefully and the residual ethanol volume in the test tube was recorded as *V*_3_. The porosity of the scaffold (e) was measured using Equation (2). The average of three measurements was taken for each sample.

(2) $$\%\;\mathrm{Porosity}=\frac{V_{1}-V_{3}}{V_{2}-V_{3}}\times 100$$

### 4.11. Degradability

The freeze-dried screws were first weighed (*W*_0_). The screws were then incubated in 10 mM PBS solution in pH = 7.4 at 37 °C for 3, 7, 14, and 21 days. The screws were weighed again using a digital scale after removing the remained water on the screw’s surface and freeze-drying. The scaffold degradation was calculated using the following equation.

(3) $$\%\;\mathrm{Degradation}=\frac{W_{0}-W_{t}}{W_{0}}\times 100$$

### 4.12. MTT

First, the screws were incubated in 70% ethanol for a day. Then, the screws (1 cm in length) were dried at room temperature and sterilized for 1 h by exposure to UV rays. The screws were then washed with sterilized PBS. MC3T3 cell lines (with a density of 1 $\times \;10^{4}$ per milliliter) were purchased from Shahid Beheshti University of Medical Sciences, Tehran, Iran. After passage, 1 × 10^4^ cells were purred on the screws by drip technique in a 96-well plate (three screws with the same length were (oblique) placed separately in three wells of a 96-well plate) and allowed to increase to 50% confluence. Then, the scaffolds were incubated for 48 h at 37 °C and 5% CO_2_ using DMEM cell culture. The medium was changed every 12 h. Then, the incubated scaffolds were transferred into a new plate. After 48 h, 10 μL of the MTT labeling reagent at the concentration of 0.5 mg/mL was added to each well and was incubated for 4 h under the same conditions (37 °C and 5% CO_2_). Then, 100 μL of the solubilization solution (DMSO) was added to each well. The plate was left to incubate at 37 °C and 5% CO_2_ overnight. The purple formazan crystals were checked and the absorbance was measured by an ELISA reader at 570 nm.

### 4.13. Cell Attachment

Cell attachment of MC3T3 cells on the screw was evaluated 48 h post cell seeding [89]. Briefly, after removing the supernatant of the screws, the cell-seeded screws were gently washed via PBS (three times). Then, the cultured MC3T3 cells (on the screw) were fixed in glutaraldehyde (2.5% *v/v*, as a fixing solution) at room temperature for 1 h. Then, glutaraldehyde was removed from cell-seeded screws and rinsed slightly via PBS. The screws were dehydrated in various concentrations of ethanol (10%, 30%, 50%, 70%, 90%, and 100%) and, finally, air-dried overnight. The samples were then analyzed by SEM.

### 4.14. DAPI Staining

In total, 4 $\times \;10^{4}$ cells were seeded on screws using the drip technique in a 24-well plate and incubated for 48 h. Then, the screws were placed in a 4% paraformaldehyde solution for 30 min and incubated at room temperature. Then, they were washed with PBS (1%) for 5 min. In the next step, 0.1% Triton solution was used for 5 min. Next, it was rinsed with PBS again, and then 5 µg/mL of 4, 6-Diamidino-2-phenylindole di-hydrochloride (DAPI, Sigma) as a nuclear stain was added to the screws and left in the dark for 5 min at room temperature, and, again, it was washed twice with PBS. It is mandatory to keep screws in the dark and PBS until fluorescent microscopy [90].

### 4.15. ALP Staining

ALP staining was performed using the ALP staining kit (Pars-Payvand, Tehran, Iran) on day 14 after MC3T3 cells were seeded on screws 7, 2 and 10. For statistical analysis and a comparison of the groups, the area of the stained zones was measured using Image J software (1.54m).

### 4.16. Alizarin Red Staining

Using the Alizarin red staining technique for calcium deposition, mineralized matrix deposition by osteo-induced BMSCs on screws 7, 2 and 10 was assessed as a marker of mature osteoblast phenotype after 21 days. To eliminate any unabsorbed dye, screws were rinsed with distilled water and then dyed with a 40 mM Alizarin red solution for 10 min at RT. Optical pictures were then obtained. For statistical analysis and to compare the groups, the area of the stained zones was measured using Image J software.

### 4.17. In Vivo Biocompatibility

In vivo biocompatibility of the screws was assessed according to previous research by Dhandapani [23]. This assessment was carried out by subcutaneous implantation of screws in male Wistar rats for evaluating the inflammatory responses. A total of 3 animals (200–250 g) were housed in a cage (25 °C, RH: 50%, 12 h/12 h night/day). The surgical procedures were approved by the Institutional Animal Ethics Committee at Islamic Azad University, Najafabad Branch (IR.IAU.NAJAFABAD.REC.1402.253). The screws were immersed in 70% ethanol overnight and then exposed to UV irradiation for 1 h. The animals were given intraperitoneal injections of xylazine (20 mg/kg body weight) and ketamine (50 mg/kg body weight) to make them unconscious. The back region of the rats was shaved and sterilized using ethanol solution (70%).

Using a sterile surgical blade, an incision (10 mm) was made on the back of animals to make a pocket. The screw was implanted underneath the skin. A non-absorbable surgical suture (Chromic, Bangalore, India) was used to close the wound. The animals were sacrificed after 26 days and tissues were collected for histopathological studies. At the end of the period, rats were euthanized using an overdose of pentobarbital (75 mg/kg) followed by CO_2_ asphyxiation. Local irritation during the period, edema, and infection were evaluated to detect cytocompatibility.

### 4.18. Statistical Analysis

Analysis of variance (ANOVA) statistical test (under the Tukey method) was employed to analyze the obtained data using DOE software (V.12) and GraphPad Prism (V.9). The results were expressed as mean ± standard deviation (SD), and *p* < 0.05 was considered a significant difference.

## Figures and Tables

**Table 1:** The governed equations and the relevant analysis of variance results.

Response	The Final Equation in Terms of Code Factors	*p*-Value	R^2^	Adj. R^2^	Pre. R^2^	AP
(CMS) ^3^	−2.017E + 5 + 4322.38A − 8637.67B	<0.0001	0.94	0.93	0.91	27.87
(BM) ^3^	−4.7E + 5 + 10244.72A − 20519.29B	<0.0001	0.94	0.93	0.90	27.44

A: PCL; B: CS; ^3^ power of the response.

**Table 2:** Results of contact angle for all screws (screw [PCL-Chi-HA]: screw1 [77.5-2.5-1.55], screw2 [95-2.5-1.9], screw3 [77.5-2.5-1.55], screw4 [77.5-2.5-1.55], screw5 [77.5-5-1.55],screw6 [77.5-0-1.55], screw7 [95-0-1.9], screw8 [60-0-1.2], screw9 [60-5-1.2], screw10 [95-5-1.9], screw11 [77.5-2.5-1.55], screw12 [77.5-2.5-1.55], screw13 [60-2.5-1.2]).

Group	PCL-Cs %	Contact Angle	Image
**Screw 1**	77.5-2.5	79.36 ± 2	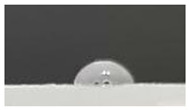
**Screw 2**	95-2.5	79.22 ± 2	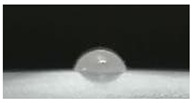
**Screw 5**	77.5-5	60.57 ± 2	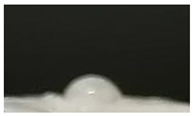
**Screw 6**	77.5-0	81.87 ± 2	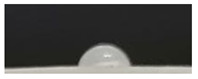
**Screw 7**	95-0	85.26 ± 2	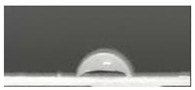
**Screw 8**	60-0	83.94 ± 2	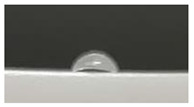
**Screw 9**	60-5	45.24 ± 2	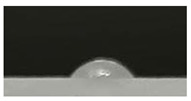
**Screw 10**	95-5	69.02 ± 2	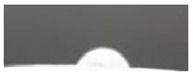
**Screw 13**	60-2.5	77.23 ± 2	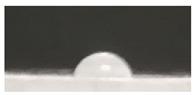

**Table 3:** Experimental design parameters and responses for PCL/chitosan-based scaffold-like screw.

Runs	Factor 2 B: PCL *w/v*%	Factor 1 A: CS *w/v*%	HA % *w/w*
1	77.5	2.5	1.55
2	95	2.5	1.9
3	77.5	2.5	1.55
4	77.5	2.5	1.55
5	77.5	5	1.55
6	77.5	0	1.55
7	95	0	1.9
8	60	0	1.2
9	60	5	1.2
10	95	5	1.9
11	77.5	2.5	1.55
12	77.5	2.5	1.55
13	60	2.5	1.2

## Data Availability

Data will be made available on request.

## References

[1] Schoon J., Hesse B., Rakow A., Ort M.J., Lagrange A., Jacobi D., Winter A., Huesker K., Reinke S., Cotte M. (2020). Metal-Specific Biomaterial Accumulation in Human Peri-Implant Bone and Bone Marrow. Adv. Sci..

[2] Al-Tamimi A.A., Fernandes P.R.A., Peach C., Cooper G., Diver C., Bartolo P.J. (2017). Metallic bone fixation implants: A novel design approach for reducing the stress shielding phenomenon. Virtual Phys. Prototyp..

[3] Mbitta Akoa D., Sicard L., Hélary C., Torrens C., Baroukh B., Poliard A., Coradin T. (2024). Role of Physico-Chemical and Cellular Conditions on the Bone Repair Potential of Plastically Compressed Collagen Hydrogels. Gels.

[4] Okazaki Y., Hayakawa E., Tanahashi K., Mori J. (2020). Mechanical Performance of Metallic Bone Screws Evaluated Using Bone Models. Materials.

[5] Lewallen E.A., Riester S.M., Bonin C.A., Kremers H.M., Dudakovic A., Kakar S., Cohen R.C., Westendorf J.J., Lewallen D.G., van Wijnen A.J. (2014). Biological Strategies for Improved Osseointegration and Osteoinduction of Porous Metal Orthopedic Implants. Tissue Eng. Part B Rev..

[6] Matassi F., Botti A., Sirleo L., Carulli C., Innocenti M. (2013). Porous metal for orthopedics implants. Clin. Cases Min. Bone Metab..

[7] Ma X.-Y., Cui D., Wang Z., Liu B., Yu H.-L., Yuan H., Xiang L.-B., Zhou D.-P. (2022). Silk Fibroin/Hydroxyapatite Coating Improved Osseointegration of Porous Titanium Implants under Diabetic Conditions via Activation of the PI3K/Akt Signaling Pathway. ACS Biomater. Sci. Eng..

[8] Arabnejad S., Johnston B., Tanzer M., Pasini D. (2017). Fully Porous 3D Printed Titanium Femoral Stem to Reduce Stress-Shielding Following Total Hip Arthroplasty. J. Orthop. Res..

[9] Al-Tamimi A.A., Peach C., Fernandes P.R., Cseke A., Bartolo P.J.D.S. (2017). Topology Optimization to Reduce the Stress Shielding Effect for Orthopedic Applications. Procedia CIRP.

[10] Ye J., He W., Wei T., Sun C., Zeng S. (2023). Mechanical Properties Directionality and Permeability of Fused Triply Periodic Minimal Surface Porous Scaffolds Fabricated by Selective Laser Melting. ACS Biomater. Sci. Eng..

[11] Agrawal C.M., Reis R.L., Cohn D. (2002). Biodegradable Polymers for Orthopaedic Applications. Polymer Based Systems on Tissue Engineering, Replacement and Regeneration.

[12] Prakasam M., Locs J., Salma-Ancane K., Loca D., Largeteau A., Berzina-Cimdina L. (2017). Biodegradable materials and metallic implants—A review. J. Funct. Biomater..

[13] Waizy H., Diekmann J., Weizbauer A., Reifenrath J., Bartsch I., Neubert V., Schavan R., Windhagen H. (2013). In vivo study of a biodegradable orthopedic screw (MgYREZr-alloy) in a rabbit model for up to 12 months. J. Biomater. Appl..

[14] Song R., Murphy M., Li C., Ting K., Soo C., Zheng Z. (2018). Current development of biodegradable polymeric materials for biomedical applications. Drug Des. Dev. Ther..

[15] Weiler A., Windhagen H.J., Raschke M.J., Laumeyer A., Hoffmann R.F.G. (1998). Biodegradable Interference Screw Fixation Exhibits Pull-Out Force and Stiffness Similar to Titanium Screws. Am. J. Sports Med..

[16] Karageorgos F.F., Alexiou M., Tsoulfas G., Alexopoulos A.H. (2024). Hydrogel-Based Vascularized Organ Tissue Engineering: A Systematized Review on Abdominal Organs. Gels.

[17] Guastaferro M., Baldino L., Reverchon E., Cardea S. (2021). Production of Porous Agarose-Based Structures: Freeze-Drying vs. Supercritical CO_2_ Drying. Gels.

[18] Sharma S., Sudhakara P., Singh J., Ilyas R., Asyraf M., Razman M. (2021). Critical review of biodegradable and bioactive polymer composites for bone tissue engineering and drug delivery applications. Polymers.

[19] Esmaeili J., Jalise S.Z., Pisani S., Rochefort G.Y., Ghobadinezhad F., Mirzaei Z., Mohammed R.U.R., Fathi M., Tebyani A., Nejad Z.M. (2024). Development and characterization of Polycaprolactone/chitosan-based scaffolds for tissue engineering of various organs: A review. Int. J. Biol. Macromol..

[20] Xiao X., Liu R., Huang Q., Ding X. (2009). Preparation and characterization of hydroxyapatite/polycaprolacton-chitosan composites. J. Mater. Sci. Mater. Med..

[21] Mad Jin R., Sultana N., Baba S., Hamdan S., Ismail A.F. (2015). Porous PCL/Chitosan and nHA/PCL/Chitosan Scaffolds for Tissue Engineering Applications: Fabrication and Evaluation. J. Nanomater..

[22] Shirzaei Sani I., Rezaei M., Baradar Khoshfetrat A., Razzaghi D. (2021). Preparation and characterization of polycaprolactone/chitosan-g-polycaprolactone/hydroxyapatite electrospun nanocomposite scaffolds for bone tissue engineering. Int. J. Biol. Macromol..

[23] Dhandapani R., Krishnan P.D., Zennifer A., Kannan V., Manigandan A., Arul M.R., Jaiswal D., Subramanian A., Kumbar S.G., Sethuraman S. (2020). Additive manufacturing of biodegradable porous orthopaedic screw. Bioact. Mater..

[24] Świerczyńska M., Król P., Hernández Vázquez C.I., Piekarska K., Woźniak K., Juszczak M., Mrozińska Z., Kudzin M.H. (2024). Blood Coagulation Activities and Influence on DNA Condition of Alginate—Calcium Composites Prepared by Freeze-Drying Technique. Mar. Drugs.

[25] Safari B., Aghanejad A., Kadkhoda J., Aghazade M., Roshangar L., Davaran S. (2022). Biofunctional phosphorylated magnetic scaffold for bone tissue engineering. Colloids Surf. B Biointerfaces.

[26] Safari B., Aghazadeh M., Aghanejad A. (2023). Osteogenic differentiation of human adipose-derived mesenchymal stem cells in a bisphosphonate-functionalized polycaprolactone/gelatin scaffold. Int. J. Biol. Macromol..

[27] Ghelich R., Jahannama M.R., Abdizadeh H., Torknik F.S., Vaezi M.R. (2019). Central composite design (CCD)-Response surface methodology (RSM) of effective electrospinning parameters on PVP-B-Hf hybrid nanofibrous composites for synthesis of HfB2-based composite nanofibers. Compos. Part B Eng..

[28] Sasikala L., Rathinamoorthy R., Dhurai B. (2018). Optimization of process conditions for chitosan-manuka honey film as wound contact layer for wound dressings. Wound Med..

[29] Gupta P., Nayak K.K. (2016). Optimization of keratin/alginate scaffold using RSM and its characterization for tissue engineering. Int. J. Biol. Macromol..

[30] Ahmadipourroudposht M., Fallahiarezoudar E., Mohd Yusof N., Idris A. (2015). Application of response surface methodology in optimization of electrospinning process to fabricate (ferrofluid/polyvinyl alcohol) magnetic nanofibers. Mater. Sci. Eng. C.

[31] Morgan E.F., Unnikrisnan G.U., Hussein A.I. (2018). Bone Mechanical Properties in Healthy and Diseased States. Annu. Rev. Biomed. Eng..

[32] Sharir A., Barak M.M., Shahar R. (2008). Whole bone mechanics and mechanical testing. Vet. J..

[33] Hart N.H., Nimphius S., Rantalainen T., Ireland A., Siafarikas A., Newton R. (2017). Mechanical basis of bone strength: Influence of bone material, bone structure and muscle action. J. Musculoskelet. Neuronal Interact..

[34] Bries A.D., Weiner D.S., Jacquet R., Adamczyk M.J., Morscher M.A., Lowder E., Askew M.J., Steiner R.P., Horne W.I., Landis W.J. (2012). A study in vivo of the effects of a static compressive load on the proximal tibial physis in rabbits. J. Bone Jt. Surgery.

[35] Choi K., Goldstein S.A. (1992). A comparison of the fatigue behavior of human trabecular and cortical bone tissue. J. Biomech..

[36] Carter D.R., Caler W.E., Spengler D.M., Frankel V.H. (1981). Uniaxial fatigue of human cortical bone. The influence of tissue physical characteristics. J. Biomech..

[37] Vikingsson L., Gómez-Tejedor J.A., Gallego Ferrer G., Gómez Ribelles J.L. (2015). An experimental fatigue study of a porous scaffold for the regeneration of articular cartilage. J. Biomech..

[38] Senatov F.S., Niaza K.V., Stepashkin A.A., Kaloshkin S.D. (2016). Low-cycle fatigue behavior of 3d-printed PLA-based porous scaffolds. Compos. Part B Eng..

[39] Wahid Z., Ariffin M., Baharudin B., Ismail M., Mustapha F. (2019). Abaqus simulation of different critical porosities cubical scaffold model. Proc. IOP Conf. Ser. Mater. Sci. Eng..

[40] Karande T.S., Ong J.L., Agrawal C.M. (2004). Diffusion in musculoskeletal tissue engineering scaffolds: Design issues related to porosity, permeability, architecture, and nutrient mixing. Ann. Biomed. Eng..

[41] Feng B., Jinkang Z., Zhen W., Jianxi L., Jiang C., Jian L., Guolin M., Xin D. (2011). The effect of pore size on tissue ingrowth and neovascularization in porous bioceramics of controlled architecture in vivo. Biomed. Mater..

[42] Tan J.C., Bennett T.D., Cheetham A.K. (2010). Chemical structure, network topology, and porosity effects on the mechanical properties of Zeolitic Imidazolate Frameworks. Proc. Natl. Acad. Sci. USA.

[43] Hallab N., Merritt K., Jacobs J.J. (2001). Metal sensitivity in patients with orthopaedic implants. JBJS.

[44] Chaya A., Yoshizawa S., Verdelis K., Myers N., Costello B.J., Chou D.-T., Pal S., Maiti S., Kumta P.N., Sfeir C. (2015). In vivo study of magnesium plate and screw degradation and bone fracture healing. Acta Biomater..

[45] Venkatesan J., Kim S.-K. (2010). Chitosan composites for bone tissue engineering—An overview. Mar. Drugs.

[46] Yao Q., Wei B., Guo Y., Jin C., Du X., Yan C., Yan J., Hu W., Xu Y., Zhou Z. (2015). Design, construction and mechanical testing of digital 3D anatomical data-based PCL–HA bone tissue engineering scaffold. J. Mater. Sci. Mater. Med..

[47] Sharifi F., Atyabi S.M., Norouzian D., Zandi M., Irani S., Bakhshi H. (2018). Polycaprolactone/carboxymethyl chitosan nanofibrous scaffolds for bone tissue engineering application. Int. J. Biol. Macromol..

[48] Chandramohan Y., Jeganathan K., Sivanesan S., Koka P., Amritha T.M.S., Vimalraj S., Dhanasekaran A. (2021). Assessment of human ovarian follicular fluid derived mesenchymal stem cells in chitosan/PCL/Zn scaffold for bone tissue regeneration. Life Sci..

[49] Schieker M., Seitz H., Drosse I., Seitz S., Mutschler W. (2006). Biomaterials as scaffold for bone tissue engineering. Eur. J. Trauma.

[50] Wan Y., Wu H., Cao X., Jeson S. (2008). Compressive mechanical properties and biodegradability of porous poly(caprolactone)/chitosan scaffolds. Polym. Degrad. Stab..

[51] Rezaei F.S., Khorshidian A., Beram F.M., Derakhshani A., Esmaeili J., Barati A. (2021). 3D printed chitosan/polycaprolactone scaffold for lung tissue engineering: Hope to be useful for COVID-19 studies. RSC Adv..

[52] Zhang X.-Y., Fang G., Zhou J. (2017). Additively manufactured scaffolds for bone tissue engineering and the prediction of their mechanical behavior: A review. Materials.

[53] Ogura K., Kanamoto T., Itoh M., Miyashiro H., Tanaka K. (1980). Dynamic mechanical behavior of chitin and chitosan. Polym. Bull..

[54] Costa-Pinto A.R., Reis R.L., Neves N.M. (2011). Scaffolds based bone tissue engineering: The role of chitosan. Tissue Eng. Part B Rev..

[55] Dwivedi R., Kumar S., Pandey R., Mahajan A., Nandana D., Katti D.S., Mehrotra D. (2020). Polycaprolactone as biomaterial for bone scaffolds: Review of literature. J. Oral. Biol. Craniofac. Res..

[56] Nawawi N., Alqap A.S.F., Sopyan I. (2011). Recent Progress on Hydroxyapatite-Based Dense Biomaterials for Load Bearing Bone Substitutes. Recent. Pat. Mater. Sci..

[57] Yedekçi B., Tezcaner A., Yılmaz B., Demir T., Evis Z. (2022). 3D porous PCL-PEG-PCL/strontium, magnesium and boron multi-doped hydroxyapatite composite scaffolds for bone tissue engineering. J. Mech. Behav. Biomed. Mater..

[58] Gerhardt L.-C., Boccaccini A. (2010). Review—Bioactive Glass and Glass-Ceramic Scaffolds for Bone Tissue Engineering. Materials.

[59] Ghosh S., Gutierrez V., Fernández C., Rodriguez-Perez M.A., Viana J.C., Reis R.L., Mano J.F. (2008). Dynamic mechanical behavior of starch-based scaffolds in dry and physiologically simulated conditions: Effect of porosity and pore size. Acta Biomater..

[60] Sadeghi-Avalshahr A., Khorsand-Ghayeni M., Nokhasteh S., Shahri M.M., Molavi A.M., Sadeghi-Avalshahr M. (2018). Effects of hydroxyapatite (HA) particles on the PLLA polymeric matrix for fabrication of absorbable interference screws. Polym. Bull..

[61] Azhar F., Olad A., Salehi R. (2014). Fabrication and characterization of chitosan–gelatin/nanohydroxyapatite–polyaniline composite with potential application in tissue engineering scaffolds. Des. Monomers Polym..

[62] Sadeghi A., Moztarzadeh F., Mohandesi J.A. (2019). Investigating the effect of chitosan on hydrophilicity and bioactivity of conductive electrospun composite scaffold for neural tissue engineering. Int. J. Biol. Macromol..

[63] Wang W., Caetano G., Ambler W.S., Blaker J.J., Frade M.A., Mandal P., Diver C., Bártolo P. (2016). Enhancing the hydrophilicity and cell attachment of 3D printed PCL/graphene scaffolds for bone tissue engineering. Materials.

[64] Kim C.H., Khil M.S., Kim H.Y., Lee H.U., Jahng K.Y. (2006). An improved hydrophilicity via electrospinning for enhanced cell attachment and proliferation. J. Biomed. Mater. Res. Part B Appl. Biomater. Off. J. Soc. Biomater. Jpn. Soc. Biomater. Aust. Soc. Biomater. Korean Soc. Biomater..

[65] Gong S.-H., Lee H., Pae A., Noh K., Shin Y.-M., Lee J.-H., Woo Y.-H. (2013). Gene expression of MC3T3-E1 osteoblastic cells on titanium and zirconia surface. J. Adv. Prosthodont..

[66] Loh Q.L., Choong C. (2013). Three-dimensional scaffolds for tissue engineering applications: Role of porosity and pore size. Tissue Eng. Part B Rev..

[67] Karageorgiou V., Kaplan D. (2005). Porosity of 3D biomaterial scaffolds and osteogenesis. Biomaterials.

[68] Shimko D.A., Shimko V.F., Sander E.A., Dickson K.F., Nauman E.A. (2005). Effect of porosity on the fluid flow characteristics and mechanical properties of tantalum scaffolds. J. Biomed. Mater. Res. Part B Appl. Biomater. Off. J. Soc. Biomater. Jpn. Soc. Biomater. Aust. Soc. Biomater. Korean Soc. Biomater..

[69] Sobral J.M., Caridade S.G., Sousa R.A., Mano J.F., Reis R.L. (2011). Three-dimensional plotted scaffolds with controlled pore size gradients: Effect of scaffold geometry on mechanical performance and cell seeding efficiency. Acta Biomater..

[70] Sun H., Mei L., Song C., Cui X., Wang P. (2006). The in vivo degradation, absorption and excretion of PCL-based implant. Biomaterials.

[71] de Britto D., Campana-Filho S.P. (2007). Kinetics of the thermal degradation of chitosan. Thermochim. Acta.

[72] Jin H., Zhuo Y., Sun Y., Fu H., Han Z. (2019). Microstructure design and degradation performance in vitro of three-dimensional printed bioscaffold for bone tissue engineering. Adv. Mech. Eng..

[73] Lam C.X., Savalani M.M., Teoh S.-H., Hutmacher D.W. (2008). Dynamics of in vitro polymer degradation of polycaprolactone-based scaffolds: Accelerated versus simulated physiological conditions. Biomed. Mater..

[74] Wang Y., Rudym D.D., Walsh A., Abrahamsen L., Kim H.-J., Kim H.S., Kirker-Head C., Kaplan D.L. (2008). In vivo degradation of three-dimensional silk fibroin scaffolds. Biomaterials.

[75] Riva R.l., Shah U., Thomassin J.-M., Yilmaz Z., Lecat A., Colige A., Jérôme C. (2019). Design of degradable polyphosphoester networks with tailor-made stiffness and hydrophilicity as scaffolds for tissue engineering. Biomacromolecules.

[76] Huang X., Lowe T.L. (2005). Biodegradable thermoresponsive hydrogels for aqueous encapsulation and controlled release of hydrophilic model drugs. Biomacromolecules.

[77] Kim G.H. (2008). Electrospun PCL nanofibers with anisotropic mechanical properties as a biomedical scaffold. Biomed. Mater..

[78] Kuo Z.K., Fang M.Y., Wu T.Y., Yang T., Tseng H.W., Chen C.C., Cheng C.M. (2018). Hydrophilic films: How hydrophilicity affects blood compatibility and cellular compatibility. Adv. Polym. Technol..

[79] Garcia Y., Collighan R., Griffin M., Pandit A. (2007). Assessment of cell viability in a three-dimensional enzymatically cross-linked collagen scaffold. J. Mater. Sci. Mater. Med..

[80] Saghafi Y., Baharifar H., Najmoddin N., Asefnejad A., Maleki H., Sajjadi-Jazi S.M., Bonkdar A., Shams F., Khoshnevisan K. (2023). Bromelain-and silver nanoparticle-loaded Polycaprolactone/chitosan Nanofibrous dressings for skin wound healing. Gels.

[81] Semnani D., Naghashzargar E., Hadjianfar M., Dehghan Manshadi F., Mohammadi S., Karbasi S., Effaty F. (2017). Evaluation of PCL/chitosan electrospun nanofibers for liver tissue engineering. Int. J. Polym. Mater. Polym. Biomater..

[82] Osathanon T., Giachelli C.M., Somerman M.J. (2009). Immobilization of alkaline phosphatase on microporous nanofibrous fibrin scaffolds for bone tissue engineering. Biomaterials.

[83] Cojocaru F.D., Balan V., Popa M.I., Lobiuc A., Antoniac A., Antoniac I.V., Verestiuc L. (2019). Biopolymers–calcium phosphates composites with inclusions of magnetic nanoparticles for bone tissue engineering. Int. J. Biol. Macromol..

[84] Seddighian A., Ganji F., Baghaban-Eslaminejad M., Bagheri F. (2021). Electrospun PCL scaffold modified with chitosan nanoparticles for enhanced bone regeneration. Prog. Biomater..

[85] Kumaraswamy H., Vinjamuri B., Rao T. (2019). Influence of Boron Fiber Powder and Graphite Reinforcements on Physical and Mechanical Properties of Aluminum 2024 Alloy Fabricated by Stir Casting. J. Miner. Mater. Charact. Eng..

[86] Azadi M., Dadashi A., Dezianian S., Kianifar M., Torkaman S., Chiyani M. (2021). High-cycle Bending Fatigue Properties of Additive-Manufactured ABS and PLA Polymers Fabricated by Fused Deposition Modeling 3D-Printing. Forces Mech..

[87] Park H., Guo X., Temenoff J.S., Tabata Y., Caplan A.I., Kasper F.K., Mikos A.G. (2009). Effect of swelling ratio of injectable hydrogel composites on chondrogenic differentiation of encapsulated rabbit marrow mesenchymal stem cells in vitro. Biomacromolecules.

[88] Cui Y., Liu Y., Cui Y., Jing X., Zhang P., Chen X. (2009). The nanocomposite scaffold of poly(lactide-co-glycolide) and hydroxyapatite surface-grafted with l-lactic acid oligomer for bone repair. Acta Biomater..

[89] Fadaie M., Mirzaei E. (2018). Nanofibrillated chitosan/polycaprolactone bionanocomposite scaffold with improved tensile strength and cellular behavior. Nanomed. J..

[90] Xing H., Yin H., Sun C., Ren X., Tian Y., Yu M., Jiang T. (2019). Preparation of an acellular spinal cord scaffold to improve its biological properties. Mol. Med. Rep..

